# Policing Relative Conflicts of Interest in Social Insects

**DOI:** 10.1371/journal.pbio.0020324

**Published:** 2004-08-24

## Abstract

Workers of social insects prevent other workers laying eggs to increase colony efficiency and not -- as traditionally thought - purely because workers are more related to the queen of the colony

The order and harmony that appears to bless the lives of many social insects, from ants to bees, has long fascinated naturalists. That individual workers seem to routinely sacrifice their own (reproductive) interests for the good of the colony has also piqued the interest of philosophers and kings, for obvious reasons. But scratch the surface and that blissful harmony reveals a complex feat of social engineering that is both exquisitely organized and potentially ruthless.

One of the altruistic behaviors that social insects are famous for is that one or a few queens perform most or all of the reproduction in a colony, while workers are, for the most part, non-reproductive. The evolution of this social structure partly stems from the unusual sex determination system of social insects, in which unfertilized eggs (of either workers or queens) develop into males and fertilized eggs (produced only by queens) develop into female queens and workers. This creates unusual relationships between family members that affect how W. D. Hamilton's theory of “kin selection” operates in these species. Kin selection, as elegantly summarized by “Hamilton's Rule,” predicts that the altruistic behavior of workers—that is, investing in the reproduction of others in the colony rather than in their own reproduction—can evolve if the indirect reproductive payoff to workers (i.e., via reproduction by relatives) is higher than the cost of the missed opportunity for direct reproduction. Kin selection revolves around relatedness because relatedness determines the magnitude of indirect reproductive payoffs. However, based on a survey of 50 species of ants, wasps, and bees, Rob Hammond and Laurent Keller now demonstrate that the behavior in the colony cannot be accounted for simply based on relatedness patterns, but that it is necessary to consider how colony efficiency influences behavior.

In some social insect colonies, workers do lay eggs, in a sense “cheating” on the other workers who are investing in the queen's reproduction rather than in their own. Such action can be severely penalized by other workers, who aggressively police the colony for the illicit offspring—or behavior—of their guilty colleagues. In honeybees, where this behavior was first shown, workers remove worker-laid eggs within hours by eating them, and, in some ants, more draconian methods lead to the mutilation of the culprit caught in the act of laying.[Fig pbio-0020324-g001]

**Figure pbio-0020324-g001:**
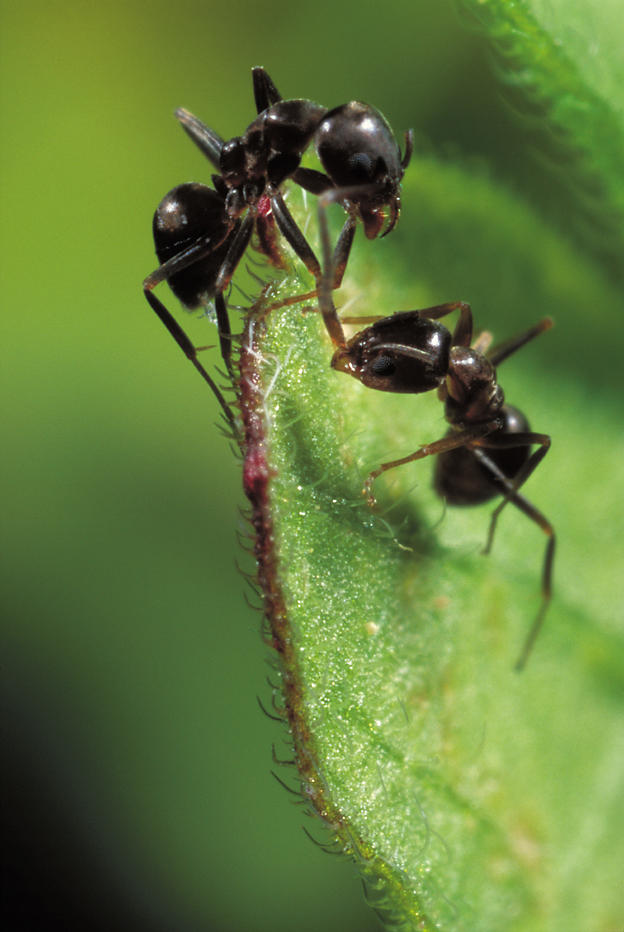
Conflict between ants (Photo: Christian König, www.konig-photo.com)

Why workers police worker reproduction in some colonies and not in others can also be influenced by relatedness. If a queen is monogamous and mates only once, then each worker will actually be more related to her nephew (produced by a sister worker) than to her brother (produced by the queen); in this case, workers should tolerate other workers' male offspring. But if the queen mates more than twice (as in honeybees) or if there are multiple queens heading a colony, then the relationship between workers becomes diluted (they do not all have the same father), and workers are more closely related to brothers than to nephews. In this case, workers should clamp down hard on any worker breeding and raise only the queen's sons (in addition to her daughters).

But workers policing the reproduction of their fellow workers could also be advantageous if the energy invested by workers into laying eggs—which would otherwise be used in foraging and legitimate brood rearing—detracts from the overall efficiency and growth of the colony. Although there is some evidence for this “efficiency hypothesis,” it is widely accepted that the driving force behind policing is primarily explained by patterns of relatedness. By doing a detailed comparative phylogenetic analysis of different species, Hammond and Keller put the “relatedness hypothesis” to the test and—contrary to expectations—found evidence that this genetic incentive for workers to police the reproduction of other workers cannot account for its widespread prevalence among social insects.

One prediction from the relatedness hypothesis is that the extent to which workers produce male offspring is determined by the relatedness of the workers. By contrast, the efficiency hypothesis predicts no such relationship. In line with this, Hammond and Keller's survey reveals that no matter how related workers are to each other, most males across this broad range of species are produced by queens. In other words, worker-policing does not depend on relatedness, so other factors—such as colony efficiency—must act as an important constraint on worker reproduction. This, Hammond and Keller emphasize, does not amount to showing that kin selection is unimportant—but it does mean that the harmony and regulation of reproduction in social insects is much more complex than expected from simple theoretical expectations based solely on relatedness.

